# Does Human Papillomavirus Infection Influence the Frequency and Severity of Nutritional Disorders in Head and Neck Cancer?

**DOI:** 10.3390/nu14214528

**Published:** 2022-10-27

**Authors:** Marcin Mazurek, Radosław Mlak, Agata Kot, Mansur Rahnama-Hezavah, Teresa Małecka-Massalska

**Affiliations:** 1 Department of Human Physiology of the Chair of Preclinical Sciences, Medical University of Lublin, 20-080 Lublin, Poland; 2 Laboratory of Body Composition Research of the Chair of Preclinical Sciences, Medical University of Lublin, 20-080 Lublin, Poland; 3 Care and Treatment Facility, Cardinal Wyszynski Voivodeship Specialist Hospital in Lublin, Biernackiego Street, 20-718 Lublin, Poland; 4 Chair and Department of Dental Surgery, Medical University of Lublin, 20-093 Lublin, Poland

**Keywords:** cachexia, inflammation, malnutrition, nutrition disorders, *Papillomaviridae*

## Abstract

Background: About 87% of head and neck cancer (HNC) patients (mostly oropharyngeal cancer—OPC) are infected with human papillomavirus (HPV). Recent studies have demonstrated a significant correlation between HPV infection and nutritional disorders in HNC patients. Therefore, we formed a hypothesis that nutritional disorders or their severity in HNC patients may be associated with the occurrence of HPV infection due to known molecular differences in involved tissue. This literature review aimed to evaluate the influence of HPV infection on the occurrence and severity of nutritional disorders in HNC patients. Materials and Methods: The PubMed database was used to search papers with the keywords “HPV”, “HNC”, and “nutritional disorders” in different variants and combinations. Conclusions: The data available in the discussed papers indicate, among other things, that HPV−positive patients may be at higher risk of malnutrition, critical weight loss, and necessity for gastrostomy after radiotherapy or chemoradiotherapy (C-RT). It should be highlighted that despite some studies demonstrating positive results, currently available data regarding the influence of HPV infection on the occurrence and severity of nutritional disorders in HNC remain limited and inconclusive, and thus further research on this issue is warranted.

## 1. Introduction

### 1.1. Nutritional Disorders

Among all disturbances of nutritional state, cachexia and sarcopenia seem to have the most serious health consequences. In head and neck cancer (HNC), nutritional disorders develop in approximately 90% of patients [1]. In turn, approximately 87% of HNC patients (mostly oropharyngeal cancer—OPC) are infected with human papillomavirus (HPV) [2]. Since all nutritional disorders have some overlapping elements and similar pathomechanisms, we focus on cancer cachexia and sarcopenia as the most serious among them.

Cachexia (wasting syndrome) is considered an irreversible, multifactorial process characterized by a progressive loss of skeletal muscle mass accompanied or not by a decrease in adipose tissue. Another commonly noted symptom is a general deterioration in mental state, which, in the case of oncological patients, often determines worse quality of life and prognosis [1]. Cachexia is commonly observed in numerous chronic diseases, especially including malignant neoplasms (e.g., HNC, gastrointestinal tumors, lung cancer) remaining one of the main causes of mortality in this group of patients [1,2].

It is estimated that in patients with an advanced stage of cancer, cachexia occurs at a frequency of 60–80%, while in 20–40%, it is considered a direct cause of death [2,3]. In about 20–30% of patients suffering from HNC, wasting syndrome is commonly diagnosed in the initial phase of treatment. In advanced HNC, wasting syndrome is observed in more than 50% of patients, and its risk increases with the severity of the disease. When we take into consideration treatment (radiotherapy—RT, chemotherapy—CTH, or chemoradiotherapy—C-RT), the percentage of patients with cachexia can even rise up to nearly 90% [4]. Moreover, cachexia has been associated with reduced CTH effectiveness and increased risk of toxic effects of therapy [1,5].

Sarcopenia is characterized by an accelerated loss of muscle mass and function. It is linked to a higher risk of falls, functional limitations, frailty, and morbidity [6,7]. Sarcopenia is frequently observed in cancer patients. Despite the fact that it could be considered a separate condition, in most cases it accompanies cachexia. In patients with gastrointestinal tumors, sarcopenia is identified in 37% of cases before surgery and in 29% of cases before CTH [7]. Sarcopenia prevalence in HNC patients ranges from 6.6% to 70.9% [8]. In cancer patients, sarcopenia is linked to more surgical infections, longer hospital stays, more chemotherapy-induced dose-limiting toxicity, and poor survival [7].

In the case of HNC, the most obvious explanation of cachexia- and/or sarcopenia-related weight loss is the location of the tumor in the head and neck region, the main site of food intake. Anatomical localization of the tumor and its surgical implications are undisputed grounds for the initiation of cachexia development; however, several other factors should not be underestimated, such as the influence of host–tumor interactions on inflammation, the endocrine system, metabolism, and many other processes [2,9,10,11,12]. It is believed that metabolic changes (increased demand for energy and/or the predominance of catabolic processes) and reduction in food intake and uptake are intensified by the ongoing inflammation [1,9,11].

**Figure 1:**
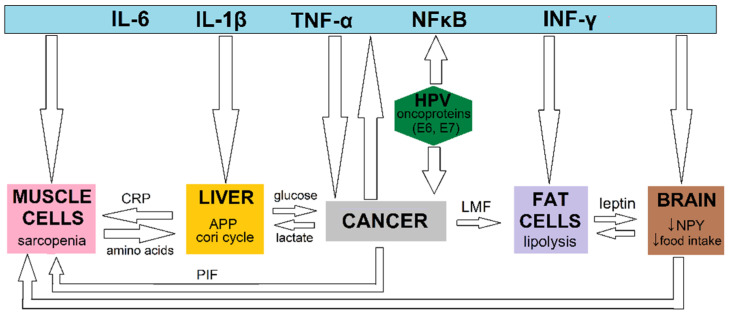
The potential impact of HPV oncoproteins on mechanisms involved in the development of nutrition disorders. Abbreviations: APP—acute phase proteins; CRP—C reactive protein; HPV—human papillomavirus; IL-1β—interleukin 1 beta; IL-6—interleukin 6; LMF—lipid mobilizing factor; NF-κB—nuclear factor kappa B; NPY—neuropeptide Y; PIF—proteolysis inducting factor; TNF-α—tumor necrosis factor alpha; ↓—decrease.

Generally, cachexia is a complex process that involves several known mechanisms. It was established that the development of cachexia is associated with an imbalance between anabolism (decreased) and catabolism (increased) in muscle and adipose tissue. In a situation where mediators participating in anabolic processes (insulin, growth hormone, insulin-like growth factor 1 (IGF-1), testosterone, and glucocorticoids) are lacking or the receptors that bind them are damaged, a predominance of catabolism over anabolism occurs [13]. In cachexia-induced catabolic processes, many different factors are involved, including inflammatory molecules (TNF-α, INF-γ, IL-6, IL-1β), hormones (cortisol, glucagon), and oxidative stress [13]. Despite the influence of TNF-α and IL-1β on appetite and metabolism, they seem to be involved in the breakdown of muscle fibers [10,11,13]. In cancer cachexia (with or without accompanied sarcopenia), this process can be explained by the simultaneous increase of proinflammatory cytokines, i.e., IL-6 and INF-γ, or TNF-α, playing an important role in the induction of ligases involved in the ubiquitin–proteasome system (UPS) activation: cytokine-dependent muscle atrophy F box (MAFbx) and muscle ring finger 1 (MuRF1). Moreover, the secretion of proinflammatory cytokines plays a key role in the regulation of the nuclear factor kappa B (NF-κB) pathway, which in turn is a crucial factor in the protein breakdown processes or the development of intracellular oxidative stress [10,14]. The relationship between HPV infection, inflammation, cancer, and host tissue and their influence on the mechanism involved in the development of nutritional disorders is presented in Figure 1.

It needs to be highlighted that in cancer patients, cachexia is commonly accompanied by sarcopenia. Therefore, its pathomechanism is in many aspects very similar to cachexia. The systemic inflammatory response has been associated with muscle wasting in cancer patients. In sarcopenia, an increased level of numerous proinflammatory cytokines, i.e., CRP, IL-6, IL-1β, and IL-8, are observed. Other mechanisms of muscle wasting include inhibition of myoblast differentiation, enhanced autophagy, and derangements in the renin–angiotensin–aldosterone system (RAAS) [15].

### 1.2. Human Papillomavirus—HPV

Human papillomavirus (HPV) belongs to the *Papillomaviridae* family and represents a group of viruses that infect the epithelial surface of the skin and mucous membranes. The WHO has classified HPV types as belonging to a high-risk group or low-risk group of cancer development [16,17,18,19,20].

Persistent HPV infection (with high-risk types) is considered the main factor leading to precancerous or cancerous transformation in several types of tissue, including the head and neck region, lower genital tract, and above all in the uterine cervix. It should be noted that only 10–15% of patients with high-grade cervical dysplasia are HPV−negative. HPV infection is responsible for about 70% of cervical cancer. Cancerous transformation is caused by precursor dysplastic lesions of the cervical epithelium. Cervical cancer is diagnosed in a significant percentage of women in developing countries. Importantly, the prognosis for patients with cervical dysplasia who receive appropriate treatment remains excellent [21,22]. Moreover, it is highlighted that HPV vaccination is the only efficacious method to reduce the risk of HPV−related cancer. The first HPV vaccine designed to prevent HPV−related cancers was approved in 2006 for women and extended in 2009 for men. All available vaccines (bivalent, quadrivalent, nonavalent) target HPV16 and HPV−18 types. To guarantee its efficacy, routine prophylactic immunization of young women (11–12 years old—vaccination should occur before sexual activity starts) should be advised [23,24,25]. Prophylactic HPV vaccinations decrease the prevalence of premalignant lesions of anogenital track, and it is suggested that they may also reduce the risk of HPV−related HNC [25].

The primary cause of cancer in about 87% of HPV−positive HNC patients is HPV16 [16]. Nearly 75% of patients with oropharyngeal squamous cell carcinoma (OPSCC) are diagnosed as HPV−positive [26]. HPV−positive head and neck squamous cell carcinoma (HNSCC), especially OPSCC tumors, are characterized by greater nodal stage, more advanced stage, more perineural invasion, and ability for extracapsular spread [27]. Interestingly, HPV−positive oropharyngeal cancer (OPC) patients have better 3-year survival compared to HPV−negative patients (82.4% vs. 57.1%). Moreover, in HPV−positive patients, the risk of death (adjusted for age, race, tumor and nodal stage, tobacco exposure, and treatment assignment) is significantly reduced (HR = 0.42; 95% CI = 0.27–0.66) [28]. It is suggested that this is due to the fact that HPV−positive patients are subjected to more intensive treatment (RTH/CTH/surgery) more frequently [27].

HPV replication processes involve eight open reading frames (ORF). The proteins encoded by these genes are divided into two groups: early proteins (E1–E7) and late proteins (L1, L2) [29,30]. The roles of E6 and E7 proteins are of great importance in the initiation of the neoplastic process by inducing changes in the differentiation and immortalization of keratinocytes, causing genomic instability and changes in the cell cycle. Increased viral particle proliferation is responsible for modifying the pathways responsible for the differentiation and division of keratinocytes, which contributes to the neoplastic transformation [16]. Integration and continuous duplication of E6 and E7 oncoproteins also result in the downregulation of p53 and Rb suppressor proteins. Oncoprotein E7 is responsible for the interaction with the Rb cellular factor, thereby interfering with the action of the E2F transcription factor through its direct binding. What is more, it was found that the E6 oncoprotein is responsible for inhibiting the transcription of the p53 protein, its degradation, and destabilization through inhibition of the activity of histone acetyltransferases. The activity of these two oncoproteins results in numerous mutations and many errors during mitosis, underlying the development of the neoplastic process [20]. On the other hand, HPV−positive cancers generally have a lower mutational burden than HPV−negative cancers, which have a high incidence of p53 mutations [26].

This literature review aimed to evaluate the potential influence of HPV infection on the frequency or severity of nutritional disorders in HNC patients.

## 2. Materials and Methods

We researched all papers published in the literature (in the PubMed database), with the following keywords: “head and neck cancer,” “HNC and HPV”, “cancer cachexia”, “sarcopenia”, “HPV infection in cancer cachexia”, “HPV immune response”, “survival in patients with HPV−related HNC”, “HNC and malnutrition”, “HPV and nutritional disorders”, “HPV and sarcopenia” and “HPV and “weight loss”. For the purposes of this review, the incidence and risk of nutritional status disorders depending on the HPV status in some cases were calculated based on the available data extracted from cited publications. Statistical analysis was performed using the odds ratio (OR) test with a 95% confidence interval (95% CI) and results were considered statistically significant at *p* < 0.05.

## 3. Relationship between HPV Infection and Nutritional Disorders in Head and Neck Cancer

### 3.1. Basic Science Information and Pathophysiology

Studies on experimental models and those focused on the molecular changes related to HPV infection seem to support a hypothesis that HPV infection is not only associated with a higher risk of malignancy but can also potentially affect the development of nutritional disorders in the course of neoplastic disease. As described by da Costa et al., mice infected with HPV16 had muscle mass loss associated with the activation of the NF-κB pathway. In the above experiment, six-week-old female K14-HPV16 mice were used. The study used hemizygous (HPV16 +/−) and wild-type (HPV16 −/−) mice. In the group that was not exposed to the rutin or curcumin, a significant decrease in muscle mass was observed in the hemizygous group compared to the wild-type variant group. The HPV16 +/− animals experienced systemic inflammation and cachexia, but whether this was due to the dysplastic lesions or HPV itself was not shown. This study lacked an HPV−negative but dysplasia-positive control, and thus the associated cachexia may not be solely attributable to HPV alone. One of the study’s main limitations was that it does not allow us to conclude that the inflammation was related to HPV infection. Indeed, in the discussion, the authors state that epidermal dysplasia (and not HPV per se) is correlated with a robust systemic inflammatory response that is known to be associated with cachexia [31].

The presence of HPV infection leading to the neoplastic transformation can be crucial for the expression and activation of the NF-κB pathway in many cancers [32]. The activation of this pathway is mainly due to E6 viral oncoprotein; however, the mechanism of this activation is not fully understood. Probably, E6 protein of high-risk HPV (HPV16, 18, and 31) is responsible for the activation of the NF-κB cascade [33]. In a study by Mishra et al., the expression of p65, p50, p52, c-Rel, RelB, and Bcl-3 proteins belonging to the NF-κB family was compared in samples derived from oral tumors of patients with or without HPV16 infection. Based on Western blot analysis, the increased expression of p50 and Bcl-3 in the group of HPV16-positive compared to HPV16-negative patients was found. Similarly, in the immunohistochemical examination, the level of p50 and Bcl-3 expression was higher in HPV16-positive tissue. Moreover, slightly higher p65 expression in HPV16-positive samples compared to those without HPV16 infection was noted [32].

The secretion of IL-1 and TNF-α can have a major impact on the regulation of the food intake process. These cytokines affect the increase of corticotrophin-releasing hormone (CRH) production, which leads to impairment in nervous regulation of food intake and modification of sensitivity to glucose. What is more, it can modulate the functioning of the digestive tract and determine nutritional satiation through the nervous system [34]. An evaluation of the change in the level of proinflammatory cytokines was performed by Kemp et al. in a group of women with confirmed HPV infection. The study included 50 women over 45 years of age with HPV infection and 50 women of similar age who were free of HPV infection. In the samples from patients with HPV infection, significantly higher levels of interleukin 8 (IL-8) (*p* < 0.0001), IL-1β (*p* < 0.0001) and TNF-α (*p* < 0.0001) were found [35].

### 3.2. Clinical Papers

Data, results, and conclusions of all publications discussed below are summarized in Table 1.

In the only currently available prospective study, conducted by Harrowfield et al., data were obtained from 83 OPSCC Caucasian patients (84.3% of patients were HPV−positive) undergoing C-RT. The patient-generated subjective global assessment (PG-SGA) was used to assess nutritional status as the primary outcome and loss of weight as the secondary outcome. At the beginning of the treatment, a lower incidence of malnutrition in HPV−positive patients was noted. On the other hand, HPV−positive patients had an insignificantly higher incidence and risk of malnutrition (B or C category) in the last week of RT (89% vs. 85%; *p* = 0.6524; OR = 1.41, 95% CI: 0.26–7.54, *p* = 0.6885) as well as one month (71% vs. 62%; *p* = 0.5184; OR = 1.56, 95% CI: 0.46–5.35; *p* = 0.4776) after treatment. Three months after treatment, HPV−positive patients had a significantly higher rate and insignificantly higher risk (42.8% vs. 38.5%; *p* = 0.0266; OR = 1.20; 95% CI: 0.36–4.04; *p* = 0.7684) of malnutrition (B or C category). Weight loss, depression, poor quality of life, and adverse events were all secondary outcomes. Additionally, in the last week of C-RT, the HPV−positive group had insignificantly lower risk of 5% weight loss (68.6% vs. 69.3%; *p* = 1.0000; OR = 0.97; 95% CI: 0.26–3.49; *p* = 0.9625). On the other hand, after 1 month (80% vs. 76.9%; *p* = 0.7238; OR = 1.20; 95% CI: 0.29–4.94; *p* = 0.8009) and 3 months (87.1% vs. 76.9%; *p* = 0.3900; OR = 2.03; 95% CI: 0.47–8.82; *p* = 0.3433) after treatment, HPV−positive patients had insignificantly higher risk of losing more than 5% of their body weight, while HPV−positive patients had insignificantly lower risk of weight loss > 10% in the last week of treatment (27.1% vs. 30.7%; *p* = 0.7476; OR = 0.84; 95% CI: 0.23–3.05; *p* = 0.7887) and 1 month after C-RT (52.8% vs. 53.8%; *p* = 1.0000; OR = 0.96; 95% CI: 0.29–3.15; *p* = 0.9477). At three months after therapy, HPV−positive patients had significantly higher risk of losing more than 10% of their body weight (67% vs. 31%; *p* = 0.0266; OR = 4.60; 95% CI: 1.28–16.52; *p* = 0.0194). Moreover, similarly to most studies, mortality after 2 years was significantly higher in HPV−positive compared to HPV−negative patients (30% vs. 7%; *p* < 0.01). The risk of weight loss and malnutrition depending on HPV status was calculated for the purposes of this study based on the extracted data [36].

Treatment-naïve HPV−positive OPSCC patients are less likely to suffer from dysphagia or tumor-induced odynophagia [37]. On the other hand, despite the reduction in RT intensity recommended, which could translate into a reduction in toxicity, currently, high-dose RT and concurrent C-RT are commonly used in these patients. This may result in both acute and late toxicity deteriorating the nutritional status, which in turn may lead to the need for percutaneous endoscopic gastrostomy (PEG). In a retrospective study by Vangelov et al., out of 100 newly diagnosed Caucasian patients with OPSCC (85% men) subjected to radical RT, 68% were HPV−positive. Among HPV−positive patients, the majority (87%) received concurrent C-RT. In 29% of HPV−positive patients, weight loss was found before the diagnosis of OPSCC. During the RT, weight loss (from 0 to 17%) was observed in almost all of the patients (except one). Additionally, compared to HPV−negative, HPV−positive patients had a significantly higher mean weight loss over time (8.4% vs. 6.1%; *p* = 0.003). Critical weight loss (CWL) was observed in 86% of patients, and the majority of them were HPV−positive (92.6%). Such factors as HPV status (positive) and C-RT were found to be predictors of CWL. Moreover, in the HPV−positive group, significantly higher risk of CWL (≥5%) (92.6% vs. 60%; OR = 8.4, 95% CI: 1.77–39.93; *p* = 0.0075) was noted. A significant percentage of HPV−positive patients required PEG (68.6%), mostly in the form of reactive feeding tubes (RFT) (43.6%). Among patients in whom feeding tube dependence (FTD) (prophylactic feeding tubes—PFT) was used, HPV−positive patients showed significantly higher mean weight loss (8.6% vs. 3.9%; *p* = 0.003). When only the HPV−positive patients were analyzed, it turned out that the patients using FTD (PFT and RFT) had significantly higher mean percentage weight loss compared to the ones in whom parenteral nutrition (PN) was not used at all (CWL in both groups, respectively: 9.6% vs. 7.1%; *p* = 0.023). HPV−positive patients had insignificantly higher risk of FT (PFT and RFT) (63.2% vs. 60%; *p* = 1.000; OR = 1.15, 95% CI: 0.29–4.46; *p* = 0.8434). Moreover, insignificantly lower risk of PFT in HPV−positive group (39.0% vs. 55.6%; *p* = 0.4641; OR = 0.51, 95% CI: 0.12–2.20; *p* = 0.3678) was noted. While patients with HPV infection had insignificantly higher risk of RFT (51.9% vs. 20%; *p* = 0.3525; OR = 4.32, 95% CI: 0.45–41.31; *p* = 0.2040). HPV−positive patients had insignificantly lower risk of CWL during PFT (41.7% vs. 60%; *p* = 0.6384; OR = 0.48, 95% CI: 0.07–3.21; *p* = 0.4460) and insignificantly higher risk of CWL during RFT (56.2% vs. 33.3%; *p* = 0.5825; OR = 2.57, 95% CI: 0.22–30.34; *p* = 0.4531). Nevertheless, the main weakness of this study is that there were only 10 HPV−negative patients (10%) as a comparison group and only 6 patients (13%) in the HPV−positive group have definitive radiation. In the case of 22 patients (22%), HPV status was unknown. The risk of weight loss and FTD depending on the HPV status was calculated for the purposes of this study based on the extracted data [38].

The influence of HPV infection on weight loss (according to the risk of prolonged feeding tube use) was analyzed by Anderson et al. in a retrospective study on 101 Caucasian patients with OPSCC. Based on the T-stage and N-stage features, the study group was divided into three subgroups: high-risk (T3 or T4 and level two lymphadenopathy: HRi, n = 28), high-intermediate risk (T3 or T4 without level two lymphadenopathy: HIRi, n = 31), and low intermediate risk (T0-T2 with level two lymphadenopathy; LIRi, n = 42) of prolonged feeding tube use. All patients were treated with definitive IMRT (with or without concurrent chemotherapy). HPV infection was confirmed in 58.4% of patients. In the study group, good adherence to PFT recommendations was noted (87%). Interestingly, in the LIRi group compared to the HRi and HIRi groups, a significantly higher proportion of HPV+ patients were noted (81% vs. 71% and 52%, respectively; *p* = 0.008). In the LIRi group, total weight loss was significantly higher compared to the HRi (8.2% vs. 4.8%; *p* = 0.002;), and HIRi (8.2% vs.5.2%; *p* = 0.006;) groups. Moreover, in the LIRi group, percentage weight loss during FT use was significantly higher compared to the HRi (HRi: 8.8% vs. 4.6%; *p* < 0.001) and HIRi (8.8% vs.5.3%; *p* = 0.002) group. However, the authors suggest that the poor nutritional outcomes in HPV−positive patients were associated with a lack of adherence to the given PFT recommendations observed in this group, resulting in suboptimal feeding tube utilization. The percentage of weight loss and weight loss during FT depending on the HPV status was calculated for the purposes of this study based on the extracted data [39].

In patients with HNC, complications related to the location of the tumor and the type of treatment are observed very often. The methods of PN include gastrostomy involving the insertion of a feeding tube into the stomach before starting treatment. The assessment of the presence of p16 in tumor tissue (surrogate of HPV16 infection) and its usefulness in the prediction of the need for gastrostomy was performed by Brown et al. in a retrospective study on 269 Caucasian patients diagnosed with oral cancer (30%), OPC (34%) and other types (36%) of HNC. Patients enrolled in the study were classified as being at high risk (88 people) or low risk (181 people) of malnutrition. In the case of 59 patients (36.2%) the HPV status was confirmed by immunohistochemistry. The risk of the necessity of gastrostomy was significantly higher in p16-positive patients (76.3% vs. 31.7%; *p* < 0.0001; OR = 4.4; 95% CI: 1.01–19.31; *p* = 0.049). The authors also cite other studies in which higher organ toxicity and dysphagia in patients with HNC who were p16 positive were noted. On the other hand, increased toxicity may explain increased rates of gastrostomy tube insertions, which in turn may suggest only an indirect association between HPV infection and malnutrition. However, a few limitations of this study need to be raised. Increased gastrostomy tube rate in p16+ patients was only in the low-risk cohort, which had a very small number of patients: only 14 (23.7%) were HPV−positive. Moreover, the authors analyzed the association of p16 status and PEG only in 19 (26.8%) patients who met the criteria for proactive gastrostomy. However, it should be highlighted that a direct comparison of malnutrition incidence by HPV status was not performed by the authors. The risk of gastrostomy depending on the HPV status was calculated for the purposes of this study based on the extracted data [40].

In contrast, Tamaki et al. conducted a retrospective study of 113 patients with OPC. HPV infection was confirmed in 85 (75.9%) patients. Among the study group, 32 (28.3%) patients had sarcopenia. The pre-treatment BMI and skeletal muscle index (SMI) values were used to divide patients as sarcopenic or nonsarcopenic. Patients with sarcopenia were more often older (63.5 vs. 57.6 years), female (76.5% vs. 53.1%), and had lower mean BMI (24.5 vs. 28.4 kg/m^2^). HPV−positive patients were characterized by significantly higher pretreatment BMI compared to HPV−negative patients (28.2 vs. 24.2 kg/m^2^; *p* = 0.001). Additionally, in the HPV−positive group, an insignificantly lower risk of sarcopenia (27.0% vs. 32.1%; *p* = 0.6332; OR = 0.78; 95% Cl: 0.31–1.98; *p* = 0.7832) was observed. Similarly to other studies, significantly lower risk of disease-free survival shortening (HR = 0.40; 95% CI: 0.20–0.81; *p* = 0.011) and overall survival shortening (HR = 0.46; 95% CI: 0.23–0.91; *p* = 0.025) were noted in HPV−positive patients. The risk of sarcopenia depending on HPV status was calculated for the purposes of this study based on the extracted data [41].

A retrospective study by Stone et al. was performed in a group of 260 Caucasian patients with HNC. Sarcopenia was diagnosed in 144 patients (55.4%). Based on the method used in the study, sarcopenia was identified when the L3 index was lower than 38.5 cm^2^/m^2^ in women and 52.4 cm^2^/m^2^ in men. The control group consisted of 116 patients with HNC and without sarcopenia. Based on the analysis of the level of p16 protein, HPV was confirmed in 92 patients (71.32%), while the absence of HPV infection was noted in 37 HNC patients (28.68%). HPV status was reported only for oropharynx cancer cases and a few oral cavity cases where p16 staining was performed. Interestingly, the presence of sarcopenia was diagnosed in 47.83% of HPV−positive patients and in 67.57% of patients without p16 protein expression (*p* = 0.0517). Therefore, there was a significantly lower risk of sarcopenia developing in HPV−positive patients when compared to the control group (OR = 0.44; 95% Cl:0.20–0.98; *p* = 0.0445). The risk of sarcopenia depending on HPV status was calculated for the purposes of this study based on the extracted data [42].

In a retrospective study performed by Olson et al., the data were obtained from 245 OPSCC Caucasian patients before treatment (RT or surgery). HPV infection was confirmed in 197 patients (87.6%). After body composition analysis (SMI), 135 patients (55.1%) were diagnosed as sarcopenic, whereas 100 patients (44.9%) were classified as nonsarcopenic. Risk of sarcopenia was insignificantly lower in HPV−positive patients (54.3% vs. 64.3%; *p* = 0.4170; OR = 0.66, 95% CI: 0.29–1.50; *p* = 0.3228). Moreover, there was no difference in overall survival between HPV−positive patients with or without sarcopenia (HR = 0.58; 95% Cl: 0.10–2.40). The limitation of this study concerns the lack of a direct comparison of the incidence of sarcopenia depending on HPV status (the risk of sarcopenia depending on HPV status was calculated for the purposes of this study based on the extracted data) [43].

A retrospective study performed by Naik et al. included 147 Caucasian patients with OPSCC who were treated with standard three-field C-RT. HPV infection was confirmed in 130 patients (88.4%). Patients with HPV−positive OPSCC at last follow-up were more likely to resume a normal diet (at 55 months after treatment start: 87% vs. 65%; *p* = 0.02), had reduced incidence of restricted diet (at 2 years after treatment start: 8.6% vs. 33.3%; *p* = 0.014) and feeding tube dependence (FTD) (at 2 years after treatment start: 1.6% vs. 12.5%; *p* = 0.06). Moreover, only HPV status was found to be a significant predictor of decreased swallowing dysfunction. In comparison to HPV−negative, HPV−positive OPSCC patients had significantly reduced risk of late (after 24 months after treatment) swallowing impairment after C-RT (HR = 0.19; 95% CI: 0.05–0.65; *p* = 0.008), while at 55 months of follow-up, HPV+ patients were more likely to resume a normal diet (87% vs. 65%; *p* = 0.0291; OR = 3.62, 95% CI: 1.19–11.09 *p* = 0.0239). The numbers, percentages, and results of statistical analysis were calculated for the purposes of this study based on the extracted data [44].

## 4. Conclusions

At some stage of the disease, nutritional disorders affect almost all patients with malignant neoplasms. This issue is extremely important in HNC patients. A pivotal role in the pathogenesis of HNC (especially OPC) is played by HPV oncoproteins (mainly E6 and E7) contributing significantly to genetic changes in host tissue and the increase in proinflammatory cytokine secretion associated with infection. On the other hand, HPV infection evades the immune system and leads to immunosuppression. However, patients with HPV−positive OPC have a better response to RT or C-RT, so this may presumably produce an increase in therapy-related inflammation with the consequent increase of inflammatory cytokines. Therefore, we are looking for an answer to the question about the potential relationship between HPV infection and the development (or severity) of nutritional disorders (especially malnutrition/cachexia and/or sarcopenia) in patients with HNC. It should be highlighted that to the best of our knowledge, to date, this is the first review systematizing knowledge about the influence of HPV infection on nutritional disorders in HNC. The HPV infection observed in patients with HNC may result in increased secretion of the same inflammation mediators, which have a key role in the development of nutritional disorders. Interestingly, before the start of treatment, BMI in HPV−positive HNC patients is usually higher. However, those patients, although rarely diagnosed with dysphagia, are characterized by higher weight loss during treatment and more often require PEG. In patients with HNC, HPV infection by changing the host genome, including the functioning of protein products of genes encoding the metabolism-modifying factors and cancer-associated inflammation can lead to malnutrition/cachexia risk change (probably increase). Unfortunately, the data in the papers quoted in this review do not provide strong evidence that HPV itself is contributing to nutritional deficiencies. In many of the studies discussed, there were no (or a very small number of) HPV−negative patients serving as a control group, and thus direct comparisons in cachexia between HPV−positive and HPV−negative patients have not been performed and we cannot make an overarching statement that HPV−positive patients have a higher risk of cachexia (or higher risk of more severe cachexia). Although treatment is a factor contributing to the development of nutritional deficiencies in this group of patients, the presence of HPV infection may reduce the risk of this process. Interestingly, the available data suggest that HNC HPV−positive patients may have a lower risk of sarcopenia. However, it should be taken into account that the available data also in this case are limited and inconclusive. Therefore, it should be further investigated what key mechanisms of development of cancer cachexia and or sarcopenia are affected by the presence of HPV oncoproteins. The knowledge acquired may in the future improve the individualization of the treatment (an early implementation of nutritional therapy, modification of the treatment regimen), which will translate into a measurable benefit for the patient. However, this requires taking into account that the risk and severity of cachexia or other nutritional disorders may be associated with the occurrence of the molecularly distinct—HPV−positive—HNC.

## Figures and Tables

**Table 1:** Studies containing data on the influence of HPV infection on the nutritional status of patients with HNC.

Reference (Year)	Race	Study Design	Nutritional Status Measure	The Time Point of Evaluation	Results				Conclusions
Harrowfield et al. (2021) [36]	Caucasian	Prospective	WL (>5%; >10%) Malnutrition (B or C according to PG-SGA)	Last week as well as 1 and 3 mths after C-RT	83 OPSCC; HPV+: 70 (84.3%); HPV−: 13(15.7%)				HPV+ patients had: (a) NS ↓ risk of >5% WL in last week C-RT (68.6% vs. 69.3%; *p* = 1.0000; OR = 0.97; 95% CI: 0.26–3.49; *p* = 0.9625). (b) NS ↑ risk of >5% WL 1 mth after C-RT (80% vs. 76.9%; *p* = 0.7238; OR = 1.20; 95% CI: 0.29–4.94; *p* = 0.8009). (c) ↑ risk of >5% WL 3 mths after C-RT (87.1% vs. 76.9%; *p* = 0.3900; OR = 2.03; 95% CI: 0.47–8.82; *p* = 0.3433). (d) NS ↓ risk of >10% WL in last week C-RT (27.1% vs. 30.7%; *p* = 0.7476; OR = 0.84; 95% CI: 0.23–3.05; *p* = 0.7887). (e) NS ↓ risk of >10% WL 1 mth after C-RT (52.8% vs. 53.8%; *p* = 1.0000; OR = 0.96; 95% CI: 0.29–3.15; *p* = 0.9477). (f) S ↑ risk of >10% WL 3 mths after C-RT (67% vs. 31%; *p* = 0.0266; OR = 4.60; 95% CI: 1.28–16.52; *p* = 0.0194). (g) NS ↑ rate and risk of malnutrition in last week of C-RT (88.6% vs. 84.6%; *p* = 0.6524; OR = 1.41; 95% CI: 0.26–7.54; *p* = 0.6885). (h) NS ↑ rate and risk of malnutrition 1 mth after C-RT (71.4% vs. 61.6%; *p* = 0.5184; OR = 1.56; 95% CI: 0.46–5.35; *p* = 0.4776). (i) S ↑ rate and NS ↑ risk of malnutrition 3 mths after C-RT (42.8% vs. 38.5%; *p* = 0.0266; OR = 1.20; 95% CI: 0.36–4.04; *p* = 0.7684).
					>5% WL (last week of C-RT) (yes): HPV+: 48 (68.6%) HPV−: 9 (69.3%)		>5% WL (last week of C-RT) (no): HPV+: 22 (31.4%) HPV−: 4 (30.7%)		
					>5% WL (1 mth after C-RT) (yes): HPV+: 56 (80%) HPV−: 10 (76.9%)		>5% WL (1 mth after C-RT) (no): HPV+: 14 (20%) HPV−: 3 (23.1%)		
					>5% WL (3 mths after C-RT) (yes): HPV+: 61 (87.1%) HPV−: 10 (76.9%)		>5% WL (3 mths after C-RT) (no): HPV+: 9 (12.9%) HPV−: 3 (23.1%)		
					>10% WL (last week of C-RT) (yes): HPV+: 19 (27.1%) HPV−: 4 (30.7%)		>10% WL (last week of C-RT) (no): HPV+: 51 (72.9%) HPV−: 9 (69.3%)		
					>10% WL (1 mth after C-RT) (yes): HPV+: 37 (52.8%) HPV−: 7 (53.8%)		>10% WL (1 mth after C-RT) (no): HPV+: 33 (47.2%) HPV−: 6 (46.2%)		
					>10% WL (3 mths after C-RT) (yes): HPV+: 47 (67%) HPV−: 4 (31%)		>10% WL (3 mths after C-RT) (no): HPV+: 23 (33%) HPV−: 9 (69%)		
					Malnutrition (last week of C-RT) (yes): HPV+: 62 (88.6%) HPV−: 11 (84.6%)		Malnutrition (last week of C-RT) (no): HPV+: 8 (11.4%) HPV−: 2 (15.4%)		
					Malnutrition (1 mth after C-RT) (yes): HPV+: 50 (71.4%) HPV−: 8 (61.6%)		Malnutrition (1 mth after C-RT) (no): HPV+: 20 (28.6%) HPV−: 5 (38.4%)		
					Malnutrition (3 mths after C-RT) (yes): HPV+: 30 (42.8%) HPV−: 5 (38.5%)		Malnutrition (3 mths after C-RT) (no): HPV+: 40 (57.2%) HPV−: 8 (61.5%)		
Olson et al. (2020) [43]	Caucasian	Retrospective	Sarcopenia (defined a priori as a skeletal muscle index of less than 52.4 for men and 38.5 for women)	Pretreatment (surgery/RT)	245 OPSCC; HPV+: 197 (87.2%); HPV−: 28 (12.8%)				HPV+ patients had NS ↓ risk of sarcopenia (54.3% vs. 64.3%; *p* = 0.4170; OR = 0.66, 95% CI: 0.29–1.50; *p* = 0.3228).
					Sarcopenia (yes): 135 HPV+: 107 (54.3%) HPV−: 18 (64.3%)		Sarcopenia (no): 110 HPV+: 90 (45.7%) HPV−: 10 (35.7%)		
Anderson et al. (2019) [39]	Caucasian	Retrospective	WL (% weight change between commencement and final week of RT). WL during FT use (as above)	Weight change between commence men and the final week of RT(6–7 weeks)	101 OPSCC; HPV+: 59 (58.4%); HPV−: 42(41.6%)				In the LIRi group: (a) compared to HRi and HIRi groups a S ↑ proportion of HPV+ patients was noted (81% vs. 71% and 52%, respectively; *p* = 0.008). (b) total WL was S ↑ compared to the HRi (8.2% vs. 4.8%; *p* = 0.002;) and HIRi (8.2% vs.5.2%; *p* = 0.006;) groups. (c) percent of WL during FT use was S ↑ compared to the HRi (HRi: 8.8% vs. 4.6%; *p* < 0.001) and HIRi (8.8% vs.5.3%; *p* = 0.002) group. (d) compared to HRi and HIRi groups a S ↑ proportion of HPV+ patients was noted (81% vs. 71% and 52%, respectively; *p* = 0.008). (e) total WL was S ↑ compared to the HRi (8.2% vs. 4.8%; *p* = 0.002;) and HIRi (8.2% vs.5.2%; *p* = 0.006;) groups. (f) percent of WL during FT use was S ↑ compared to the HRi (HRi: 8.8% vs. 4.6%; *p* < 0.001) and HIRi (8.8% vs.5.3%; *p* = 0.002) group.
					% of WL:				
					LIRi: 8.2%	HRi: 4.8%		HIRi: 5.2%	
					% of WL during FT use:				
					LIRi: 8.8%	HRi: 4.6%		HIRi: 5.3%	
Tamaki et al.(2019) [41]	Caucasian	Retrospective	BMI (pretreatment) Sarcopenia Male: SMI < 43 cm^2^/m^2^ and BMI < 20.0 kg/m^2^ (underweight) or 20.0–24.9 kg/m^2^ (normal weight) SMI <41 cm^2^/m^2^ and BMI = 25.0–29.9 kg/m^2^ (overweight) and BMI = 30.0 kg/m^2^ (obese) Female: SMI < 41 cm^2^/m^2^ and all BMI categories	Pretreatment (surgery, C-RT and/or adjuvant treatment)	113 OPC; HPV+: 85 (75.9%); HPV−: 27 (24.1%)				HPV+ patients had: (a) S ↑ pretreatment BMI (28.2 vs. 24.2 kg/m^2^; *p* = 0.001). (b) NS ↓ risk of sarcopenia (27.0% vs. 32.1%; *p* = 0.6332; OR = 0.78; 95% Cl: 0.31–1.98; *p* = 0.7832).
					Sarcopenia (yes): 32 HPV+: 23 (27%) HPV−: 9 (32.1%)		Sarcopenia (no): 81 HPV+: 62 (73%) HPV−: 19 (67.9%)		
Stone et al. (2019) [42]	Caucasian	Retrospective	Sarcopenia (defined as L3 skeletal muscle index below 52.4 cm^2^/m^2^ for men and below 38.5 cm^2^/m^2^ for women	Pretreatment (surgery)	260 HNC; HPV+: 92 (71.3%); HPV−: 37(28.7%)				HPV+ patients had S ↓ risk of sarcopenia (47.8% vs. 67.6%; *p* = 0.0517; OR = 0.44; 95% Cl:0.20–0.98; *p* = 0.0445).
					Sarcopenia (yes): 144 HPV+: 44 (47.8%) HPV−: 25 (67.6%)		Sarcopenia (no): 116 HPV+: 48 (52.2%) HPV−: 12 (32.4%)		
Vangelov et al. (2018) [38]	Caucasian	Retrospective	CWL (defined as ≥5% WL during treatment) FTD (PFT, RFT)	In the 1st and in 6th week of RT	100 OPSCC; HPV+: 68 (87.2%); HPV−: 10 (12.8%)				HPV+ patients had: (a) S ↑ risk of CWL (92.6% vs. 60%; *p* = 0.011; OR = 8.4, 95% CI: 1.77–39.93; *p* = 0.0075). (b) ↑ risk of FT used (PFT and RFT) (63.2% vs. 60%; *p* = 1.000; OR = 1.15, 95% CI: 0.29–4.46; *p* = 0.8434). (c) NS ↓ risk of PFT (39.0% vs. 55.6%; *p* = 0.4641; OR = 0.51, 95% CI: 0.12–2.20; *p* = 0.3678). (d) NS ↑ risk of RFT (51.9% vs. 20%; *p* = 0.3525; OR = 4.32, 95% CI: 0.45–41.31; *p* = 0.2040). (e) NS ↓ risk of CWL during PFT (41.7% vs. 60%; *p* = 0.6384; OR = 0.48, 95% CI: 0.07–3.21; *p* = 0.4460). (f) NS ↑risk of CWL during RFT (56.2% vs. 33.3%; *p* = 0.5825; OR = 2.57, 95% CI: 0.22–30.34; *p* = 0.4531).
					CWL (yes): 86 HPV+: 63 (92.6%) HPV−: 6 (60%)		CWL (no): 14 HPV+: 5 (7.4%) HPV−: 4 (40%)		
					FTD (PFT, RFT) (yes): 49 HPV+: 43 (63.2%) HPV−: 6 (60%)		FTD (PFT, RFT) (no): 29 HPV+: 25 (36.8%) HPV−: 4 (40%)		
					PFT (yes): 21 HPV+: 16 (39%) HPV−: 5 (55.6%)		PFT (no): 29 HPV+: 25 (61%) HPV−: 4 (44.4%)		
					RFT (yes): 28 HPV+: 27 (51.9%) HPV−: 1 (20%)		RFT (no): 29 HPV+: 25 (48.1%) HPV−: 4 (80%)		
					PFT + CWL (yes): 18 HPV+: 15 (41.7%) HPV−: 3 (60%)		PFT + CWL (no): 23 HPV+: 21 (58.3%) HPV−: 2 (40%)		
					RFT + CWL (yes): 28 HPV+: 27 (56.2%) HPV−: 1 (33.3%)		RFT + CWL (no): 23 HPV+: 21 (43.8%) HPV−: 2 (66.7%)		
Brown et al. (2017) [40]	Caucasian	Retrospective	High-risk category: proactive gastrostomy placement before treatment: C-RT or severe malnutrition (defined as >10% WL in 6 mths, BMI < 20 with unintentional WL 5–10% in 6 mths; PG-SGA C)	From baseline at diagnosis to the end of treatment (surgery/C-RT/RT)	269 HNC; p16+: 59 (36.2%); p16-: 104 (63.8%)				HPV+ patients had S ↑ risk of gastrostomy (76.3% vs. 31.7%; *p* < 0.0001; OR = 4.4; 95% CI: 1.01–19.31; *p* = 0.049).
					↑ risk of gastrostomy: 88 p16+: 45 (76.3%) p16-: 33 (31.7%)		↓risk of gastrostomy:181 p16+: 14 (23.7%) p16-: 71 (68.3%)		
Naik et al. (2015) [44]	Caucasian	Retrospective	Diet changes (significant restrictions in the types of foods eaten, and/or requiring nutritional supplementation for weight maintenance) FTD Swallowing disorders (defined as FTD or limited diet)	3, 6, 12, and 24 mths after C-RT	147 OPSCC; HPV+: 130 (88.4%); HPV−: 17 (11.6%)				HPV+ patients had: (a) S ↓incidences and risk of restricted diet (8.6% vs. 33.3%; *p* = 0.014; OR = 0.19, 95% CI: 0.05–0.06; *p* = 0.0082) (b) S ↓ risk of FTD (1.6% vs. 12.5%; *p* = 0.06; OR = 0.11, 95% CI: 0.01–0.85; *p* = 0.0338) at last follow-up (2 years after treatment start). (c) S reduced risk of late (24 mths) swallowing impairment after C-RT (HR = 0.19; 95% CI: 0.05–0.65; *p* = 0.008). (d) were S more likely to resume a normal diet at 55 mths of follow-up (87% vs. 65%; *p* = 0.0291; OR = 3.62, 95% CI: 1.19–11.09 *p* = 0.0239).
					Restricted diet (24 mths after C-RT) (yes): HPV+: 11 (8.6%) HPV−:5 (33.3%)		Restricted diet (24 mths after C-RT) (no): HPV+:117 (91.4%) HPV−:10 (67.7%)		
					FTD (24 mths after C-RT) (yes): HPV+: 2 (1.6%) HPV−: 2 (12.5%)		FTD (24 mths after C-RT) (no): HPV+: 127 (98.6%) HPV−: 14 (87.5%)		
					Resume a normal diet (at 55 mths of follow-up) (yes): HPV+: 113 (87%) HPV−: 11 (65%)		Resume a normal diet (at 55 mths of follow-up) (no): HPV+: 17 (13%) HPV−: 6 (35%)		

Numbers, percentages and results of statistical analysis were extracted directly from publications or calculated based on available data. Abbreviations: CI—confidence interval; C-RT—chemoradiotherapy; CWL—critical weight loss; FTD—feeding tube dependence; HIRI—high-intermediate risk; HNC—head neck cancer; HPV—human papillomavirus virus; HRi—high risk; HR—hazard ratio; LIRi—low-intermediate risk; mth—month; mths—months; NS—non-significant; OPC—oropharyngeal carcinoma; OPSCC—oropharyngeal squamous cell carcinoma; OR—odds ratio; PFT—prophylactic feeding tube; PG-SGA—patient generated-subjective global assessment; RFT—reactive feeding tube; RT—radiotherapy; S—significantly; SMI—skeletal muscle index; WL—weight loss; ↑—higher; ↓—lower.

## Data Availability

Not applicable.
